# The SSU Processome Component Utp25p is a Pseudohelicase

**DOI:** 10.17912/micropub.biology.000606

**Published:** 2022-09-22

**Authors:** Rafe Helwer, J. Michael Charette

**Affiliations:** 1 Department of Chemistry, Brandon University, Brandon, Manitoba, Canada.; 2 Children’s Hospital Research Institute of Manitoba, Winnipeg, Manitoba, Canada.; 3 CancerCare Manitoba Research Institute, Winnipeg, Manitoba, Canada.

## Abstract

RNA helicases are involved in nearly all aspects of RNA metabolism and factor prominently in ribosome assembly. The SSU processome includes 10 helicases and many helicase-cofactors. Together, they mediate the structural rearrangements that occur as part of ribosomal SSU assembly. During the identification of the SSU processome component Utp25/Def, it was noticed that the protein displays some sequence similarity to DEAD-box RNA helicases and is essential for growth. Interestingly, mutational ablation showed that Utp25’s DEAD-box motifs are dispensable. Here, we show that the Utp25 AlphaFold prediction displays considerable structural similarity to DEAD-box helicases and is the first fully validated pseudohelicase.

**Figure 1. The structure of Utp25 is similar to DEAD-box RNA helicases.**
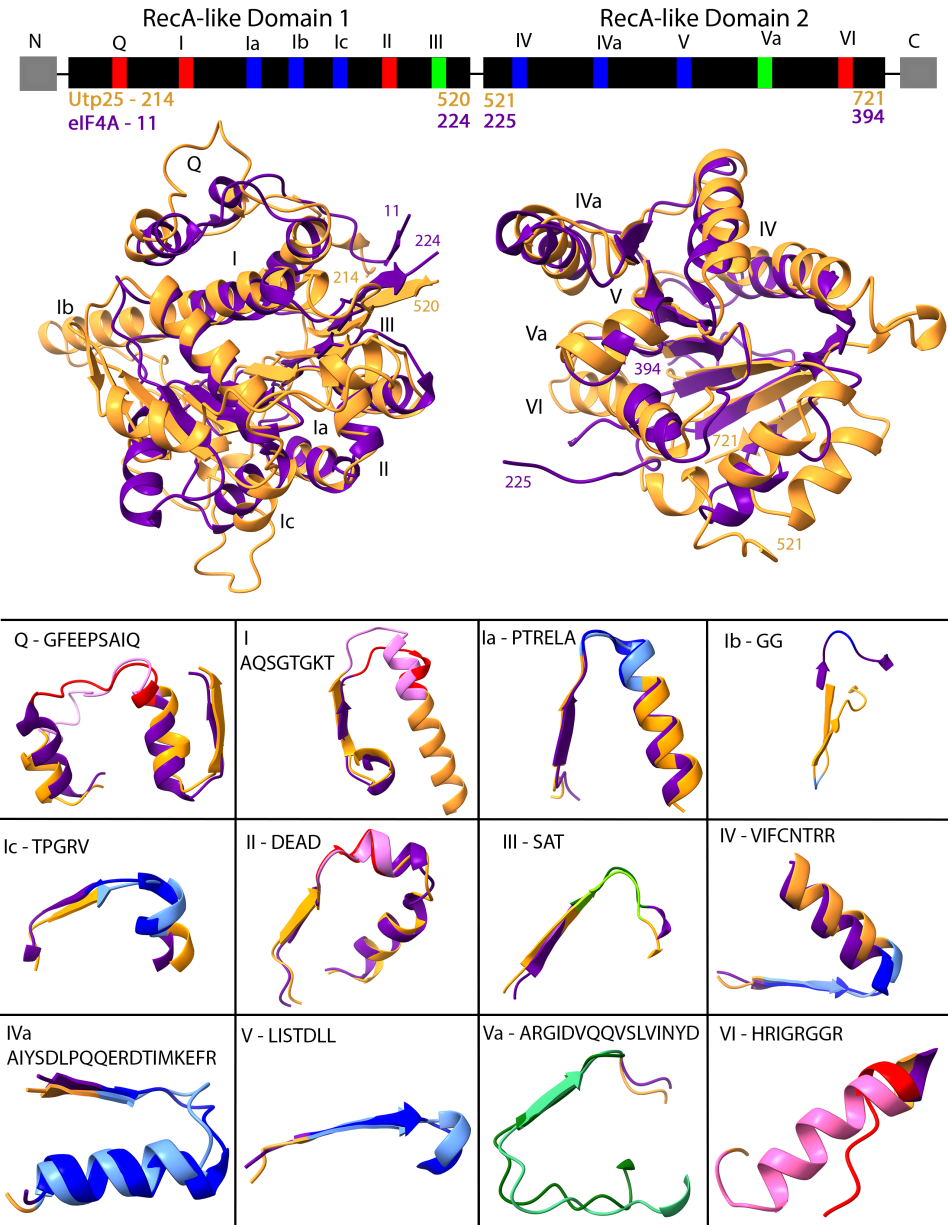
**(Top)** Linear arrangement of the DEAD-box family motifs, with sequences mediating RNA binding in blue, ATP binding and hydrolysis in red, and those linking ATP and RNA binding in green (adapted from Fig 1a; (Putnam and Jankowsky 2013)). Accessory sequences, shown in grey, are often found at the N- and C-termini and are believed to mediate protein-protein or protein-RNA interactions (Linder and Jankowsky 2011). For Utp25, accessory sequences are located at the N-terminus (Extended figure 1 and Fig S1 in (Charette and Baserga 2010)). **(Middle)** Structural alignments of domains 1 (N-term, left) and domains 2 (C-term, right) of yeast Utp25 (gold; AlphaFold P40498) and eIF4A (purple; 1FUU; (Caruthers *et al* . 2000)) show that each domain is superimposable, with a slight variation in the location of a few loops and helices. The location of the DEAD-box family motifs is indicated along with the coordinates of the regions used in the individual structural alignments of domains 1 and of domains 2, as in the top panel. **(Bottom)** High structural overlap of individual DEAD-box motifs. The colour of the DEAD-box motifs is derived from the top panel with Utp25 in the lighter colour (such as pink) and eIF4A in the darker colour (such as red). Secondary structures flanking the DEAD-box motifs are coloured as in the middle panel. The DEAD-box motif sequences (based on (Putnam and Jankowsky 2013)) are from the yeast eIF4A.

## Description

RNA helicases comprise a large family of proteins involved in nearly all aspects of RNA metabolism, from ribosome assembly and translation, to intron splicing and viral replication (Rocak and Linder 2004). The functions of RNA helicases include: unwinding and annealing RNA duplexes, clamping and dissociation of RNA-protein interactions, and chaperoning ribonucleoprotein (RNP) assembly and remodelling (Bourgeois *et al* . 2016).

The DEAD-box family of RNA helicases is a subgroup of the superfamily 2 (SF2) of helicases that contain their namesake DEAD or DEAH amino acid sequence. They are conserved proteins with both RNA-binding and ATPase functions, and are further defined by the presence of 12 conserved protein sequence motifs (Figure 1-Top; Q, I, Ia, Ib, Ic, II, III, IV, IVa, V, Va, and VI) that mediate RNA binding and ATP binding and hydrolysis (Naineni *et al* . 2022). DEAD-box helicases assemble into two lobed structures, with motifs Q-III and IV-VI each forming a Recombinase A-like (RecA-like) domain. This results in the presence of an open conformation that becomes closed upon ATP and RNA binding (Theissen *et al* . 2008).

There are at least 20 RNA helicases involved in yeast ribosome assembly, all of which are part of the DEAD-box family, with one exception (Bernstein *et al* . 2006; Granneman *et al* . 2006; Rodríguez-Galán *et al* . 2013). While their exact functions are not always known, they are believed to mediate the structural rearrangements that occur in ribosome assembly. This includes rRNA folding and unfolding, unwinding of rRNA duplexes, disruption of rRNA/protein and rRNA/snoRNA interactions, and the remodelling of intermediary rRNA structures (Woolford and Baserga 2013; Mitterer and Pertschy 2022).

RNA helicases factor prominently in the small subunit (SSU) processome, a large ribonucleoprotein involved in the pre-rRNA processing, maturation, and assembly of the small subunit of the ribosome (Dragon *et al* . 2002; Phipps *et al* . 2011). The SSU processome includes at least 10 helicases (out of ~78 proteins), along with many known and putative helicase-cofactors (Vincent *et al* . 2018).

Surprisingly, unlike other enzymes - and despite their RNA-binding activity - helicases show minimal sequence or structure specificity, likely due to their interaction with the RNA backbone (see references in (Naineni *et al* . 2022)). Instead, sequence specificity may be provided through an RNA-binding protein cofactor that recruits the helicase to that region, through protein-protein interactions, and stimulates its ATPase activity (Silverman *et al* . 2003; Rodríguez-Galán *et al* . 2013). This may additionally confer temporal and spatial specificity to helicases (Sloan and Bohnsack 2018). Cofactor mediated specificity was first shown for the helicase Dbp8 and its cofactor Esf2, both SSU processome components (Granneman *et al* . 2006).

Utp25 was originally identified as a digestive-organ expansion factor (Def) in zebrafish (Chen *et al* . 2005). It was then further characterised by the labs of Susan Baserga (Charette and Baserga 2010) and Carla Oliveira (Goldfeder and Oliveira 2010) through their search for additional components of the SSU processome. In searching through a set of yeast nucleolar proteins of unknown function, Utp25 was identified as an essential conserved protein with limited sequence similarity to DEAD-box RNA helicases. Closer inspection of Utp25 revealed motif Ia along with partial motif Q, IV, V, and VI sequences (see Fig 2 in (Charette and Baserga 2010)). However, mutational ablation of motifs Ia and VI had no discernible effect on growth. The absence of other helicase motifs, such as the DEAD sequence, suggested that Utp25 is no longer a functional helicase (see evolutionary discussion below). However, the function of Utp25 in the SSU processome was not determined. Here, we show that despite considerable sequence and functional divergence, the Utp25 AlphaFold prediction (Jumper *et al* . 2021; Varadi *et al* . 2022) is structurally similar to DEAD-box RNA helicases (Figure 1). Thus, we propose that Utp25 is a pseudohelicase, a catalytically inactive helicase that remains essential for ribosome assembly.

With the recent availability of AlphaFold structures (Jumper *et al* . 2021; Varadi *et al* . 2022), we asked if the SSU processome component Utp25/Def (Charette and Baserga 2010; Goldfeder and Oliveira 2010), which contains vestigial DEAD-box sequence motifs, adopts a structure similar to that of helicases and could thus be a pseudohelicase.

As there are no experimentally determined Utp25 structures, we first evaluated the predicted AlphaFold structure of the yeast Utp25 (P40498). The protein’s N-terminal region (aa 1 to ~160) contains repetitive sequence elements (Charette and Baserga 2010) and is predicted to be an intrinsically disordered region (IDR; Extended figure 1). Unsurprisingly, the AlphaFold predicted structure of this region possesses a very low to low per-residue confidence score (pLDDT) of <70, consistent with AlphaFold predictions of IDRs (Akdel *et al* . 2021). The structured region (aa ~160 to 721) consists of two domains with a predicted confident to very high confident score (pLDDT >70; Extended figure 2). The overall pLDDT score for the full-length yeast Utp25 structure is 82.1. The high local and overall pLDDT scores suggest a high confidence in the AlphaFold predicted yeast Utp25 structure. We further evaluated the AlphaFold yeast Utp25 structure (P40498) by aligning it to the AlphaFold human UTP25/DEF structure (Q68CQ4) using ChimeraX (Petterson *et al.* 2021). Our results suggest that the yeast and human Utp25s are highly similar (Extended figure 3) over their structured central and C-terminal domains (over 425 residues, corresponding to the RecA-like Domains 1 and 2), with a root-mean-square deviation (RMSD) of 0.993 Å (excluding IDR regions (Extended figure 1-Top and Bottom); RMSD of 19.932 Å over all residues). Thus, the sequence of Utp25 is conserved (see Figure S1 in (Charette and Baserga 2010)), along with its structure (Extended figure 1 and 3).

We then used the AlphaFold predicted structure of yeast Utp25 (P40498) as a query in a Dali search (Holm 2020) of the RCSB Protein Data Bank (Burley *et al* . 2021) to find other proteins with similar, experimentally determined, structures. The top hit in our Dali search (Extended figure 4), with a Z score of 17.4, was to the partial structure of the yeast eukaryotic initiation factor 4A (eIF4A nucleotide-binding domain; Tif2; 1QDE), with the top 158 hits to members of the helicase family from various organisms. A similar Dali search (Holm 2020) using the AlphaFold structure of the human UTP25/DEF (Q68CQ4) also identified other helicases. We then used ChimeraX (Petterson *et al.* 2021) to align the AlphaFold yeast Utp25 (P40498) to that of the highest scoring structure of yeast eIF4A containing both domains (Z score of 17.3; Tif2; 1FUU_B; (Caruthers *et al* . 2000)). Our initial alignment of the entire Utp25 and eIF4A proteins found good structural overlap of one or the other of the two RecA-like domains but not of both domains at the same time (Extended figure 5). We propose that this is due to the conformational flexibility of helicases, with the open and closed conformation (Theissen *et al* . 2008) affecting the positioning of the two RecA-like domains relative to each other. We thus isolated the domains by introducing a break in the helicase hinge region and individually aligned the N- and C-terminal RecA-like domains. In the alignment, the RMSD for domain 1 was 9.719 Å (Extended figure 6) and for domain 2 was 6.460 Å (Extended figure 7). In highly superimposable regions, the RMSD was 1.122 Å (over 90 residue pairs) and 1.204 Å (over 63 residue pairs) for domains 1 and 2, respectively (Figure 1-Middle). Each of the helicase motifs (Figure 1-Bottom) is individually superimposable except for motif Ib. Interestingly, the similarity of sequences flanking the conserved helicase motifs (see Fig 2A in (Charette and Baserga 2010)) is seen as superimposable secondary structure elements flanking the helicase motifs.

Here, we propose that Utp25 is the first fully validated pseudohelicase. This is based on Utp25 being an essential protein, having vestigial but non-functional helicase motifs (Charette and Baserga 2010), and adopting a conserved helicase structure (Figure 1). We draw a distinction between Utp25 as a catalytically dead helicase and what other groups have called a “pseudo-helicase” or “putative helicase” (Schwarz *et al* . 2013; Stanley *et al* . 2006), which is actually a catalytically active translocase that couples ATP hydrolysis to directional movement on nucleic acid strands (Singleton *et al* . 2007).

Pseudoenzymes (so called “zombie enzymes”) were first observed in kinases, where it is believed that a gene duplication and divergence event resulted in a kinase that lost the ability to phosphorylate substrates but retained an essential role, such as a regulatory function (Kwon *et al* . 2019). Subsequently, other pseudoenzymes have been discovered including pseudophosphatases, pseudoproteases, pseudonucleases, pseudosulfotransferases, and others (Ribeiro *et al* . 2019). Pseudoenzymes have so far been observed as having 4 different functions: as allosteric activators, competitive inhibitors, signalling switches, and as protein scaffolds in complex assembly (Mace and Murphy 2021). Generally, pseudoenzymes are identified and classified based on sequence analysis (with a single residue change being enough to inactivate catalytic ability), structural analysis, or functional assay (such as assessing ATP hydrolysis), with a combination of the last two criteria being considered the gold standard (Ribeiro *et al* . 2019). In proposing that Utp25 be classified as a pseudoenzyme, we are relying on published sequence and functional/mutational analysis (Charette and Baserga 2010; Goldfeder and Oliveira 2010) and the structural investigation presented here. Thus, Utp25 meets all the requirements for being a pseudoenzyme.

To our knowledge, Utp25 is only the second proposed pseudohelicase and the first pseudoenzyme component of the SSU processome. The first identified pseudohelicase, PriA in *Dinococcus radiodurans* (Lopper *et al* . 2015), based the classification on variant helicase sequences and functional assays that found that PriA has a negligible ability to unzip DNA or to catalyse ATP hydrolysis but is capable of binding to ssDNA, DNA replication forks, and to the DnaB helicase. The structure of the *D. radiodurans* PriA pseudohelicase has not been determined and its mechanism of action, along with the overall effect of PriA depletion on growth, remains unknown.

In light of Utp25 being a pseudohelicase, what might its role be in the SSU processome? One option is that Utp25 is a helicase-cofactor, binding to (pre-)rRNA and recruiting an SSU processome helicase to that site. Utp25 has been shown by co-immunoprecipitation to bind to (pre-)rRNA, either by itself or as part of a complex (Charette and Baserga 2010; Goldfeder and Oliveira 2010). In addition, Utp25 forms direct protein-protein interactions with other known or suspected helicase co-factors such as Esf2, Lcp5, Pfa1, and Slx9, and more importantly with the helicase Dhr2 (Vincent *et al* . 2018), thus identifying the helicase(s) it potentially regulates. In support of the transient nature of helicase/co-factor interactions in the SSU processome, recent cryoEM structures of the *Saccharomyces cerevisiae* , *Chaetomium thermophilum* , and human SSU processomes (which presumably consist of stably-associated components) do not include any helicases and only 2 helicase-cofactors (Barandun *et al* . 2017; Cheng *et al* . 2017; Singh *et al* . 2021). Hence the absence of Utp25 in the cryoEM structures is consistent with it being a helicase co-factor, a regulatory role which is a known function of pseudoenzymes. As with other pseudoenzymes, Utp25 may also play a protein scaffolding role in the assembly of the SSU processome, as demonstrated by the 84 KDa protein (in yeast) migrating in a glycerol gradient as a 165 kDa particle (Goldfeder and Oliveira 2010; Vincent *et al* . 2018).

In addition to being consistent with the previously proposed evolutionary appearance of Utp25 through gene duplication and divergence (Charette and Baserga 2010), our findings also increase our understanding of protein evolution. A larger and better characterised set of pseudoenzymes, to now include a pseudohelicase category, allows for a more precise estimation of the rate and pattern of amino acid change. In turn, this may yield more accurate predictions of where mutations are likely to occur within proteins. This has the potential to better inform our predictions of the consequences of protein sequence variants in genetic diseases and in cancer.

Thus, we present the first structural analysis of a pseudohelicase, showing that Utp25 fulfils all three criteria for classification as a pseudoenzyme: sequence and function (Charette and Baserga 2010; Goldfeder and Oliveira 2010), along with structure (shown here). Still unknown is whether Utp25 is capable of directly binding to (pre-)rRNA and its exact mechanism within the SSU processome.

## Methods

IDRs were predicted using the Predictor of Natural Disordered Regions (PONDR-VLXT; http://www.pondr.com/ ). The AlphaFold-predicted structures of the yeast (P40498, F1-model v2) and human (Q68CQ4) Utp25 were obtained from the AlphaFold Protein Structure Database ( https://alphafold.ebi.ac.uk/ (Jumper *et al* . 2021; Varadi *et al* . 2022)). The predicted Utp25 structures were used in a Dali search ( http://ekhidna2.biocenter.helsinki.fi/dali/ (Holm 2020)) to identify proteins with similar, experimentally determined, structures in the RCSB Protein Data Bank (PDB; rcsb.org ; (Burley *et al* . 2021)). Molecular graphics, analyses, and structural alignments were performed with UCSF ChimeraX 1.3 (Resource for Biocomputing, Visualization, and Informatics, UCSF, NIH P41-GM103311 (Pettersen *et al* . 2021)).

## Extended Data

Description: Extended figure 1: The N-terminal region of the yeast and human Utp25 is disordered. (Top) PONDR prediction of an N-terminal IDR (aa 1 to ~160) in the yeast Utp25. (Bottom) PONDR prediction of an N-terminal IDR (aa 1 to ~185) in the human UTP25. Resource Type: Image. DOI: 10.22002/D1.20292

Description: Extended figure 2: The high pLDDT domains 1 and 2 of the AlphaFold yeast Utp25 align well to eIF4A. Structural alignment of the individual RecA-like domains 1 and 2 of the AlphaFold yeast Utp25 (P40498) and yeast eIF4A (1FUU; (Caruthers et al. 2000)) crystal structure. The AlphaFold yeast Utp25 is coloured based on pLDDT score, from very low confidence in red to very high confidence in blue (key in bottom right) whereas the yeast eIF4A crystal structure is shown in brown. In domain 1, six regions in the AlphaFold yeast Utp25 are coloured cyan (70 < pLDDT < 90; confident) as opposed to blue (pLDDT > 90, very high confidence). Two of these areas overlap with helicase motifs Q and Ib. Similarly, there are 7 confident areas in domain 2 (cyan), with one overlapping helicase motif VI. The remainder of the helicase motifs are in high confidence areas. The location of the helicase motifs is indicated along with the coordinates of the regions used in the structural alignments of isolated domains 1 and 2. Resource Type: Image. DOI: 10.22002/D1.20293

Description: Extended figure 3: The yeast and human AlphaFold Utp25 structures are conserved. ChimeraX file of the aligned yeast (P40498) and human (Q68CQ4) AlphaFold Utp25 structures shows that they are clearly superimposable and thus conserved in structure. Resource Type: InteractiveResource. DOI: 10.22002/D1.20294

Description: Extended figure 4: Dali search using the yeast and human AlphaFold Utp25 structures robustly identifies known DEAD-box helicases. Resource Type: Dataset. DOI: 10.22002/D1.20295

Description: Extended figure 5: Alignment of the full-length Utp25 and eIF4A structures. ChimeraX file of the aligned full-length (containing RecA domains 1 and 2) AlphaFold yeast Utp25 (P40498) and yeast eIF4A (1FUU; (Caruthers et al. 2000)) crystal structure. Note the structural similarity of domains 1 of Utp25 and of eIF4A and similarly of domains 2 of both proteins. Structural overlap is only possible for one or the other of the two RecA-like domains but not for both domains simultaneously. Resource Type: InteractiveResource. DOI: 10.22002/D1.20296

Description: Extended figure 6: Alignment of the RecA-like domains 1 of Utp25 and of eIF4A. ChimeraX file of the aligned domains 1 of the AlphaFold yeast Utp25 (P40498) and yeast eIF4A (1FUU; (Caruthers et al. 2000)) crystal structure. Resource Type: InteractiveResource. DOI: 10.22002/D1.20297

Description: Extended figure 7: Alignment of the RecA-like domains 2 of Utp25 and of eIF4A. ChimeraX file of the aligned domains 2 of the AlphaFold yeast Utp25 (P40498) and yeast eIF4A (1FUU; (Caruthers et al. 2000)) crystal structure. Resource Type: InteractiveResource. DOI: 10.22002/D1.20298
